# FGF6 promotes cardiac repair after myocardial infarction by inhibiting the Hippo pathway

**DOI:** 10.1111/cpr.13221

**Published:** 2022-03-30

**Authors:** Zhicheng Hu, Peng Chen, Linlin Wang, Yu Zhu, Gen Chen, Yunjie Chen, Zhenyu Hu, Lin Mei, Weijing You, Weitao Cong, Litai Jin, Xu Wang, Yang Wang, Xueqiang Guan

**Affiliations:** ^1^ Department of Cardiology The Second Affiliated Hospital and Yuying Children's Hospital of Wenzhou Medical University Wenzhou P.R. China; ^2^ School of Pharmaceutical Science Wenzhou Medical University Wenzhou P.R. China; ^3^ Children's Heart Center, Institute of Cardiovascular Development and Translational Medicine The Second Affiliated Hospital and Yuying Children's Hospital of Wenzhou Medical University Wenzhou P.R. China; ^4^ Department of Histology and Embryology Institute of Neuroscience, Wenzhou Medical University Wenzhou P.R. China; ^5^ College of Pharmacy Chonnam National University Gwangju South Korea; ^6^ Department of Pharmacy Ningbo first Hospital Ningbo PR China; ^7^ School of Medical Technology Ningbo College of Health Sciences Ningbo PR China

## Abstract

**Objectives:**

Myocardial infarction (MI) commonly occurs in patients with coronary artery disease and have high mortality. Current clinical strategies for MI still limited to reducing the death of myocardial cells but failed to replace these cells. This study aimed to investigate the role of fibroblast growth factor 6 (FGF6) in enhancing the proliferative potential of cardiomyocytes (CMs) after ischemic injury via the Hippo pathway.

**Materials and Methods:**

Expression of FGF6 protein was analysed in mice with MI induced by ligation of the left anterior descending coronary artery. Activation of the Hippo pathway and the proliferation potential were examined in ischemic CMs, treated with FGF6 protein or transfected with an adeno‐virus carrying *FGF6* sh‐RNA. Immunofluorescence staining and western blotting were performed to assess the relationship between FGF6 and the Hippo pathway.

**Results:**

We found that FGF6 expression was significantly increased in the MI mouse model. Knockdown of FGF6 synthesis resulted in poorer heart function after MI. By contrast, treatment with recombinant human FGF6 protein improved heart function, reduced infarct size, and promoted cardiac repair. Additionally, FGF6 restrains the activation of the Hippo pathway and subsequently promotes nuclear accumulation of YAP. This was largely counteracted by treatment with extracellular signal‐regulated kinase 1/2 (ERK1/2) inhibitor U0126.

**Conclusion:**

FGF6 inhibits the Hippo pathway via ERK1/2, and facilitates nuclear translocation of YAP, and thereby promotes cardiac repair after MI.

## Graphical abstract

Schematic illustration of the protective effects of FGF6 on cardiomyocytes under ischemic conditions. Myocardial infarction would cause the loss of a huge number of cardiomyocytes. In this study, we revealed that FGF6 restrains the Hippo pathway via ERK, and promotes YAP nuclear translocation then increase cardiomyocytes proliferation and cardiac regeneration.

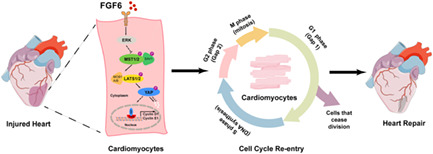

## 1 INTRODUCTION

MI is one of the most common cardiac vascular diseases worldwide. Due to that MI causes loss of many CMs, consequently, morbidity and mortality rates following MI remain high.^1^ Interventional therapy is the most common and standard care for patients with MI. Although the number of patients with fatal MI has declined, interventional therapy cannot replace cell death upon MI.^2^, ^3^ As a results, the loss of CMs eventually leads to a decline in left ventricular (LV) function and contributes to an increase in the prevalence of heart failure.^4^, ^5^ The inability to replace lost CMs is the largest obstacle in the treatment of MI. To overcome this, tremendous interest in strategies that increase the number of CMs by stimulating re‐entry of CMs into the cell cycle, survival of CMs via inhibition of apoptosis, and differentiation of CMs from stem cells have been explored.^6^, ^7^ Nevertheless, current clinical strategies for MI reduce the death of myocardial cells, but fail to replace these cells.^7^ Therefore, novel strategies to promote cell cycle re‐entry in CMs must be developed.

Regenerative therapies are conceptually different, because they aim to rebuild the myocardium rather than salvage tissue.^8^ In contrast to organs such as the liver and skin, the heart possesses only a minimal regenerative capacity.^9^, ^10^, ^11^ It lacks a progenitor cell population, and CMs exit the cell cycle shortly after birth and few of these cells re‐enter the cell cycle after injury.^12^, ^13^ Thus, any loss of CMs is essentially irreversible and can lead to or exaggerate heart failure, which is a major public health problem.^14^, ^15^ New therapeutic options are urgently needed; however, regenerative therapies in cardiovascular medicine are only in their infancy.

The fibroblast growth factor (FGF) family consists of 23 members, which can promote cell proliferation.^16^ For example, FGF6 is a critical component of the muscle regeneration process in mammals.^17^ It belongs to a family of cytokines that controls cell proliferation, cell differentiation, and morphogenetic events. Several lines of evidence demonstrate that FGF6 exhibits a restricted expression profile and is predominantly expressed in the myogenic lineage.^18^, ^19^ Floss et al. found that FGF6 can completely restore experimentally damaged skeletal muscle,^20^ leading us to speculate that it may elicit similar in the heart, which is a muscle tissue too. To investigate the effect of FGF6 on MI, we first examined its expression in the heart after permanent ligation of the left anterior descending coronary artery and confirmed that FGF6 expression was increased in mice with MI. Then, we investigated the therapeutic effect of FGF6 on MI and found that FGF6 alleviated MI by promoting cardiac repair.

Acting as a major downstream effector of the Hippo signalling pathway, YAP plays a critical role in controlling organ size.^21^ Accumulating evidence demonstrates the detrimental role of the Hippo/YAP pathway in regulating the division of CMs and the regeneration of damaged myocardium through multiple transcriptional mechanisms.^22^, ^23^

Accordingly, in this study, we demonstrated that the cardioprotective effect of FGF6 is mainly mediated by the Hippo/YAP pathway and that FGF6 triggers cell cycle re‐entry of CMs and promotes myocardial repair via this pathway. Our observations raise the possibility that targeting FGF6 can facilitate CMs cell cycle re‐entry and promote cardiac repair after injury in mammals.

## 2 METHOD

### 2.1 Animals and procedures

For animal models of MI, male C57BL/6 mice at the age of 6–7 weeks were purchased from Wenzhou Medical University, Licence No. SCXK[ZJ]2005‐0019. All experimental procedures for animal studies were approved by the ethics committee of Wenzhou Medical University and performed in accordance with the Guide for the Care and Use of Laboratory Animals. Animals were housed at 23 ± 2°C with humidity of 50 ± 5%. Mice were randomly divided into five groups (*n* >5 for each group): sham group, MI group, MI + FGF6 group, MI+ Ad‐cTNT‐sh‐*FGF6* group, and MI + FGF6 + verteporfin (VP) group. MI was established in male mice. The adult mice received tracheal intubation and were ventilated with 3% isoflurane for induction and 2% isoflurane for maintenance of anaesthesia. Then, the left anterior descending coronary artery was ligated with 7–0 prolene suture, and 25 μL Ad‐cTNT‐sh‐*FGF6* (8.69 × 10^10^ PFU/mL) or Ad‐cTNT‐sh‐Con was injected into the myocardium following MI. After completion of the surgery, the chest was closed. Mice were then warmed for several minutes until recovery. The mice in the recombinant human FGF6 protein (10 μg/kg/day, R&D, 238‐F6‐025) treated‐group were intraperitoneally administered into mice once a day for 2 weeks, while mice in the PBS group were injected with PBS buffer. VP (20 mg/kg/day, MCE, 129497‐78‐5) was intraperitoneally administered into mice once a day for 1 week, other groups was injected corn oil once a day for 1 week before sacrifice. After 2 weeks of MI, the mice were anaesthetized and sacrificed for collection of heart tissues and follow‐up examinations.

### 2.2 Neonatal rat cardiomyocytes (NRCMs) isolation, culture and transfection

Neonatal rat cardiomyocytes (NRCMs) was isolated from 1 to 3‐day‐old Sprague–Dawley rats. NRCMs was seeded in six‐well plates coated with gelatin at a density of 5 × 10^5^ cells per well and cultured in DMEM (Gibco, C11995500BT) medium supplemented with 10% foetal bovine serum (Gibco, 16000‐044) and 1% penicillin/streptomycin at 37°C with 5% CO_2_. For OGD model, structure NRCMs was cultured in no glucose DMEM (Gibco, 11966‐025) medium 6 h at 37°C with 1% O_2_, 94% N_2_, 5% CO_2_. For RNA interference, transient transfection of small interfering RNA targeting FGF6 (Si‐*FGF6*) (ORIGENE, SR507934) or a control scramble (Si‐Scr) (ORIGENE, SR507934) in NRCMs was performed using Lipofectamine™ 2000 Transfection Reagent (Thermo Fisher Scientific, 11668019) according to the manufacturer's instructions. NRCMs was infected with Si‐*FGF6* (10 nM) or Si‐Scr (10 nM) in a Opti‐MEM™ (Thermo Fisher Scientific, 31985‐070) medium for 24 h then treatment with OGD 6 h. For FGF6 protein treatment group, NRCMs was performed using recombinant human FGF6 protein (10 ng/mL, R&D, 238‐F6‐025) to the DMEM (Gibco, 11966‐025) medium without glucose then treatment with OGD 6 h. For inhibitors use, NRCMs was performed using recombinant human FGF6 protein (10 ng/mL, R&D, 238‐F6‐025) and U0126 (30 μM, MCE, HY‐12031) or VP (1 μM, MCE, HY‐B0146) to the no glucose DMEM (Gibco, 11966‐025) medium then treatment with OGD 6 h.

### 2.3 Western blot and antibodies

The equal amounts (30 μg) of protein lysed from heart tissue and cardiomyocytes, were separated by SDS‐PAGE and then transferred onto polyvinylidene fluoride (PVDF) membranes (Merck Millipore, IPVH00010). Next, membranes were blocked with 5% BSA in Tris‐buffered saline containing 0.1% (vol./vol.) Tween 20 (TBST) for 1 h at room temperature and probed with primary antibodies against corresponding antigens overnight at 4°C. Then, membranes were incubated with appropriate secondary antibodies, HRP‐goat‐anti‐mouse (Abcam, ab6789) or HRP‐goat‐anti‐rabbit (Abcam, ab6721), to bind the primary antibodies for 1 h at room temperature. The proteins were visualized by exposure machine (GE, Amersham Imager 680) using Pierce™ ECL Plus Western Blotting Substrate (Thermo Fisher Scientific, 32132) and the protein bands were quantitatively analysed with ImageJ software.

The primary antibodies used are as follows: FGF6 (Santa Cruz, sc‐373,927), YAP (CST, 14074), p‐YAP (CST, 13008), p‐ERK1/2 (CST, 9101), ERK1/2 (CST, 9102), p‐LATS1 (CST, 4668), LATS1 (Abcam, ab243656), p‐MST1 (CST, 49332), MST1 (CST, 14946), Cyclin D1 (CST, 55506), Cyclin E1 (CST, 20808), PCNA (CST, 13110), CTGF (Abcam, ab6992), and CYR61 (CST, 39382, Abcam, ab228592). β‐Actin (CST, 4970) was used as the internal reference to normalized protein expression levels.

### 2.4 TTC stain

After MI, the animals were anaesthetized and intubated, and the chest was opened. Hearts were excised, and LVs were sliced into 1‐mm‐thick cross sections. The heart sections were then incubated with a 1% TTC (Aladdin, T130066‐5 g) solution at 37°C for 15 min. The infarct area (white), and total LV area from both sides of each section were measured using ImageJ software (NIH), and the values obtained were averaged. The percentages of each section infarct area were multiplied by the weight of the section and then totalled from all sections. Infarct area/LV were expressed as percentages.

### 2.5 Echocardiographic analysis

Echocardiographic examination was performed after MI or Sham surgery for 2 weeks. The mice were firstly anaesthetized with isoflurane (1–1.5% for maintenance) mixed in 1 L/min O_2_ via a facemask, and meanwhile maintained normal breathing. Next, the parameters of cardiac function were evaluated by long‐axis M‐mode echocardiography using small animal ultrasound system (Vevo 2100, Canada) with a linear 30‐MHz transducer as described in detail. The LV ejection fraction (EF) and fractional shortening (FS), the indicators of cardiac function, were calculated and averaged from at least three consecutive cardiac cycles. All of these measurements were performed by a single experienced technician in a blinded manner.

### 2.6 Immunofluorescence

Heart section (5 μm) were subject to deparaffinization and rehydration followed by antigen retrieval by heating the slides in 10 mM Citrate buffer (pH 6.0) at 95°C for 10 min. For cardiomyocytes, NRCMs cultured on glass coverslips were fixed in paraformaldehyde for 15 min at room temperature. After washing for three times with PBS each for 5 min, cardiomyocytes and heart sections were permeabilized with 0.5% (vol./vol.) Triton X‐100 for 15 min and washing for three times with PBS each for 5 min then blocked with 5% (vol./vol.) bovine serum albumin (BSA, Sigma‐Aldrich, B2064) for 1 h at room temperature. Next, the samples were incubated primary antibodies at 4°C overnight. After washing, samples incubated secondary antibodies. Finally, DAPI was used to label the cell nuclei. Images were acquired with a Leica SP8 confocal microscopy (Leica TCS SP8, Wetzlar, Germany).

The primary antibodies used are as follows: FGF6 (Santa Cruz, sc‐373927), c‐TNT (ABclonal, A4914), Ki‐67 (D3B5) Rabbit mAb (Alexa Fluor® 647 Conjugate) (CST, 12075), YAP (CST, 14074), Aurora B (Abcam, ab2254).

The secondary antibodies used are as follows: Goat Anti‐Mouse IgG H&L (Alexa Fluor® 488) (Abcam, ab150113), Goat Anti‐Mouse IgG H&L (Alexa Fluor® 647) (Abcam, ab150115), Goat Anti‐Rabbit IgG H&L (Alexa Fluor® 488) (Abcam, ab150077), Donkey Anti‐Rabbit IgG H&L (Alexa Fluor® 647) (Abcam, ab150075).

### 2.7 EdU assay

For NRCMs, the no glucose medium was replaced with no glucose medium containing 10 μM EdU (Beyotime, C0075) 3 h after OGD, and cells were fixed 3 h later. For EdU assay adult mice cardiomyocytes, a dose of EdU (50 mg/kg/day, Beyotime, ST067) per animal was injected intraperitoneally once a day for 3 days. For EdU staining, the heart sections and cells were incubated with Click**™** EdU 555 Imaging Kit reagents (Beyotime, C0075) to reveal EdU incorporation according to the manufacturer's instructions.

### 2.8 Histological analysis

Heart tissue was fixed in 4% paraformaldehyde solution for 24 h and embedded in paraffin after dehydration using 70%–100% ethanol. The heart paraffin blocks were cut transversely into 5 μm sections. After deparaffinization, the heart sections subjected to Masson's trichrome staining were performed according to instruction book. To determined myocyte cross‐sectional area, heart sections were stained with Alexa Fluor 488 conjugated wheat germ agglutinin (WGA) (WGA‐Alexa488, Thermo Fisher Scientific, W11261) for 1 h to demarcate the cell boundaries. After staining, images were acquired with a confocal laser scanning microscope (Leica TCS SP8, Wetzlar, Germany). The myocyte cross‐sectional area and LV collagen volume were quantitatively measured by ImagePro Plus software version 7.0 (Media Cybernetics, Rockville, MD).

### 2.9 Real‐time quantitative PCR

Total RNA was extracted from heart tissue and cardiomyocytes using TRIzol reagent (Takara Bro Inc, 9108), as described by the manufacturer's instructions. The RNA samples (1 ng) was reversely transcribed to cDNA by the Hiscript® III Reverse Transcriptase kit (Vazyme, R223‐01). RT‐qPCR analysis was performed on a QuantStudio™ 3 Real‐Time PCR Detection System using ChamQ Universal SYBR qPCR Master Mix (Vazyme, Q711‐02) with specific primers. The relative expression levels of each gene were quantitated using the 2^−∆∆CT^ method and normalized to the amount of endogenous glyceraldehyde‐3‐phosphate dehydrogenase (GAPDH). The sequences of specific primers used for RT‐PCR in this study are listed in Table S1.

### 2.10 Statistical analysis

Data were analysed by GraphPad Prism 8.0 and results were presented as mean ± standard error of the mean (S.E.M.). Differences of each sample were evaluated using the unpaired Student's two‐tailed *t* test or analysis of variance (ANOVA). If *p* value ≤0.05, the result was considered significant different. All experiments were repeated at least three times.

## 3 RESULTS

### 3.1 FGF6 expression is increased after MI

**FIGURE 1:**
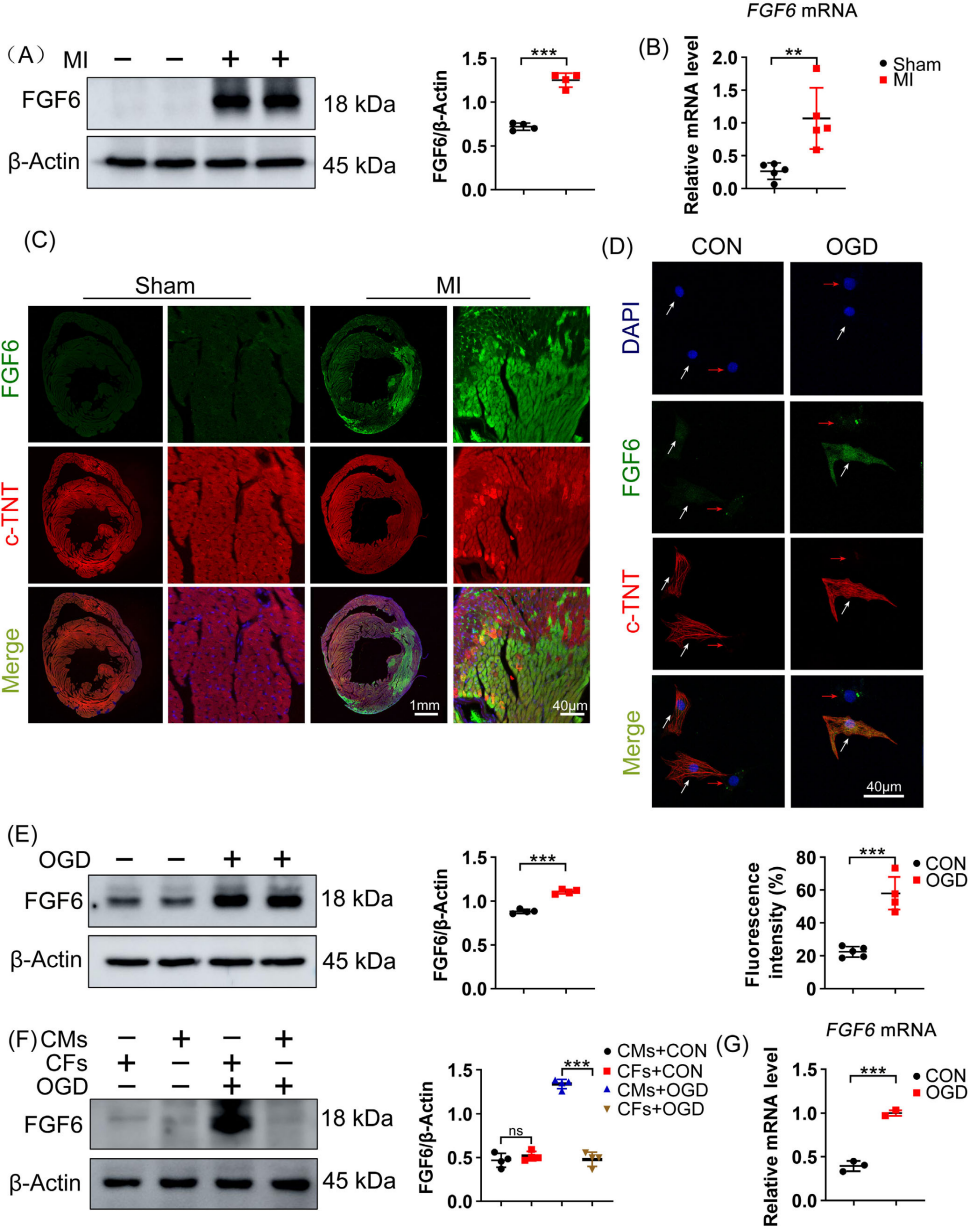
FGF6 expression is increased after MI. (A) Western blot was performed and quantitatively analysed to determine the protein levels of FGF6 in the hearts from MI (2 weeks) or sham‐operated controls. *n* = 4 per group. (B) RT‐PCR analysis of the mRNA levels of *FGF6* in the hearts of MI (2 weeks) or sham. *n* = 5 per group. (C) Representative immunofluorescence staining analysis of FGF6 proteins in the heart tissues from MI (2 weeks) or sham. Scale bar =40 μm. (D) Representative immunofluorescence and quantitative analysis of FGF6 proteins in NRCMs with or without OGD (no glucose, 1% O_2_) for 6 h, white arrow indicates CMs, red arrow indicates CFs. *n* = 5 per group. Scale bar =40 μm. (E) Western blot was performed and quantitatively analysed to determine the protein levels of FGF6 in NRCMs with or without OGD (no glucose, 1% O_2_) for 6 h. *n* = 4 per group. (F) Western blot was performed and quantitatively analysed to determine the protein levels of FGF6 in NRCMs and NRCFs. *n* = 4 per group. (G) RT‐PCR analysis of the mRNA levels of *FGF6* in NRCMs with or without OGD (no glucose, 1% O_2_) for 6 h. *n* = 3 per group. Data represent means ±SEM. Two‐tailed Student's *t* test. ***p <0.001

To explore the change in FGF6 expression after MI, we first examined the level of FGF6 in hearts of mice subjected to permanent ligation of the left anterior descending coronary artery for 2 weeks or to a sham operation. Western blotting showed that FGF6 expression was significantly increased after MI (Figure 1A), especially, FGF6 was mainly expressed at infarct area (IA) rather than border zone (BZ) or remote area (RA) (Figure S1A). Meanwhile, RT‐PCR analysis demonstrated that MI significantly increased the mRNA level of *FGF6* (Figure 1B). Consistently immunofluorescence staining showed that FGF6 expression was upregulated in hearts after MI and from CMs (Figure 1C), and this results was further confirmed it (Figure S1B). Similarly, in vitro immunofluorescence staining of cardiac troponin T (c‐TNT) the CMs marker, and FGF6 demonstrated that OGD mainly elevated expression of FGF6 in primary neonatal rat cardiac myocytes (NRCMs), but not in other cell type (Figure 1D). At the same time we found FGF6 was markedly up‐regulated in NRCMs after 6 h of oxygen–glucose deprivation (OGD) treatment as well (Figure 1E). In addition, immunofluorescence results and western blotting demonstrated that OGD mainly elevated expression of FGF6 in NRCMs, not in cardiac fibroblasts. Nevertheless, the expression of FGF6 was pretty low in both cells under normal conditions (Figure 1F). In parallel, the mRNA level of *FGF6* significantly increased after OGD treatment (Figure 1G). Thus, FGF6 was specifically upregulated in CMs after MI.

### 3.2 FGF6 alleviates heart injury in the MI mouse model

**FIGURE 2:**
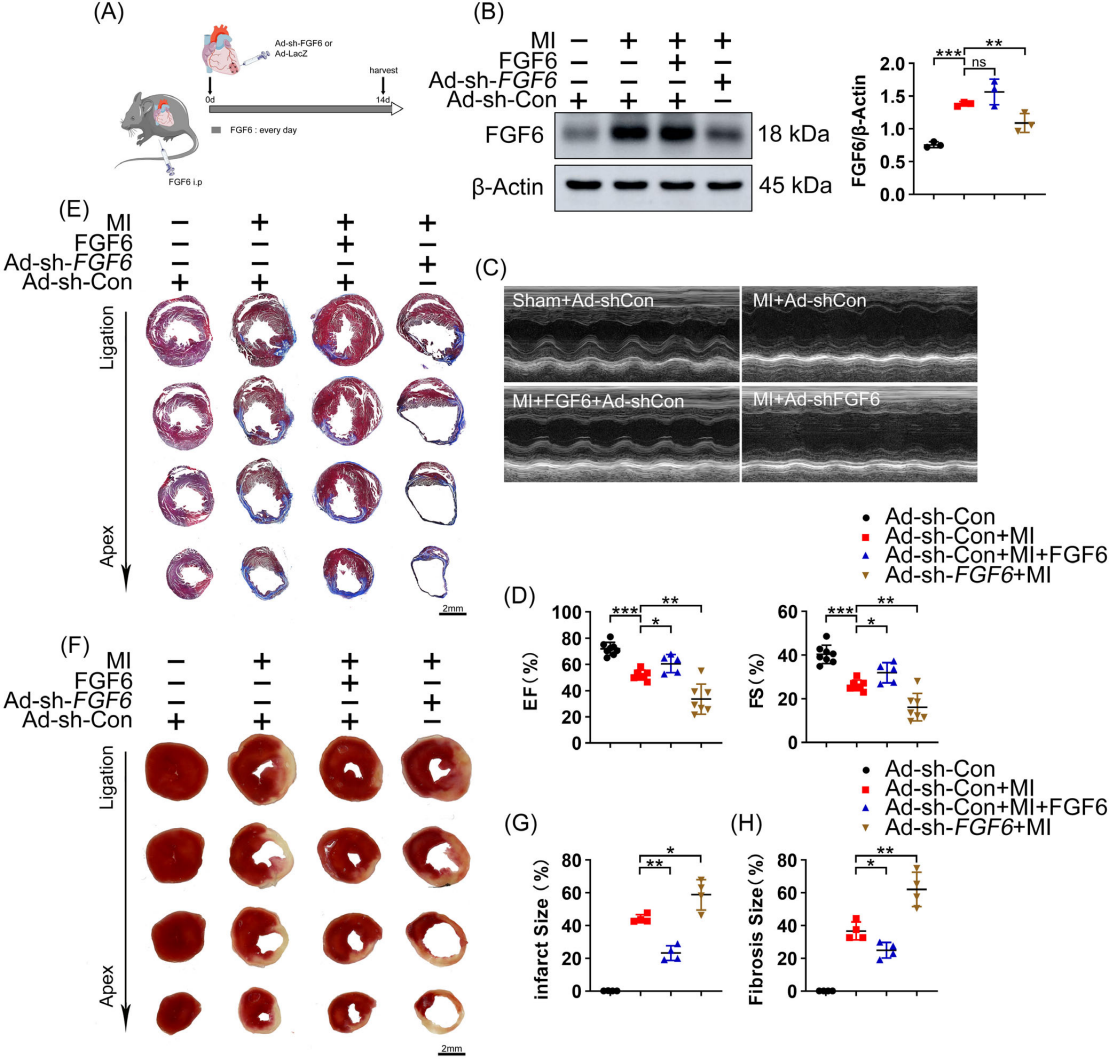
FGF6 ameliorates MI‐induced cardiac dysfunction. (A) Schematic diagram demonstrates the animal experiment design. (B) Western blot was performed and quantitatively analysed to determine the protein levels of FGF6 in the hearts of different group mice. (C) Representative M‐mode echocardiographic recording obtained from different groups mice. *n* >5 per group. (D) Quantitative analysis of LV ejection fraction (EF), fractional shortening (FS). (E) Masson trichrome staining was performed to detect cardiac fibrosis area of different group mice. Scale bar = 2 mm. *n* = 4 per group. (F) TTC staining was performed to detect cardiac ischemic area of different group mice. Scale bar = 2 mm. *n* = 4 per group. (G) Quantitative analysis of cardiac ischemic area in (F). (H) Quantitative analysis of cardiac fibrosis area in (E). Data represent means ± SEM. Two‐tailed Student's *t* test. *p <0.05, **p <0.01, ***p <0.001

FGF6 reportedly plays a vital role in muscle regeneration. However, its function in cardiac disease is unclear. To investigate this, MI mice were intraperitoneally injected with recombinant human FGF6 protein (10 μg/kg) daily for 2 weeks. The other groups were injected with an adeno‐virus carrying *FGF6* sh‐RNA (Ad‐sh‐*FGF6*) or control sh‐RNA (Ad‐sh‐Con) driven by the CM‐ specific cTNT promoter into the ischemic area of the heart after ligation of the left anterior descending artery. Echocardiography was performed to investigate cardiac function after 2 weeks treatment (Figure 2A), and the knockdown efficiency of Ad‐sh‐*FGF6* was analysed by western blotting (Figure 2B). As expected, echocardiography showed that 2 weeks FGF6 treatment improved cardiac function, as reflected by increases of the ejection fraction (EF) and fractional shortening (FS) in comparison with control MI mice (Figure 2C,D). On the contrary, echocardiography demonstrated that Ad‐sh‐*FGF6* worsened cardiac function (Figure 2C,D).

To further verify the important role of FGF6 in MI, Masson's and TTC staining were performed. This revealed that mice injected with FGF6 protein had a smaller infarct size and fibrotic area, while mice injected with AD‐sh‐*FGF6* exhibited more scarring and increased fibrotic deposition (Figure 2E–H). Together, these results suggest that recombinant human FGF6 protein alleviates heart injury in the MI mouse model, while knockdown of FGF6 aggravates heart dysfunction.

### 3.3 FGF6 promotes the cardiomyocytes cell cycle re‐entry after ischemic injury

**FIGURE 3:**
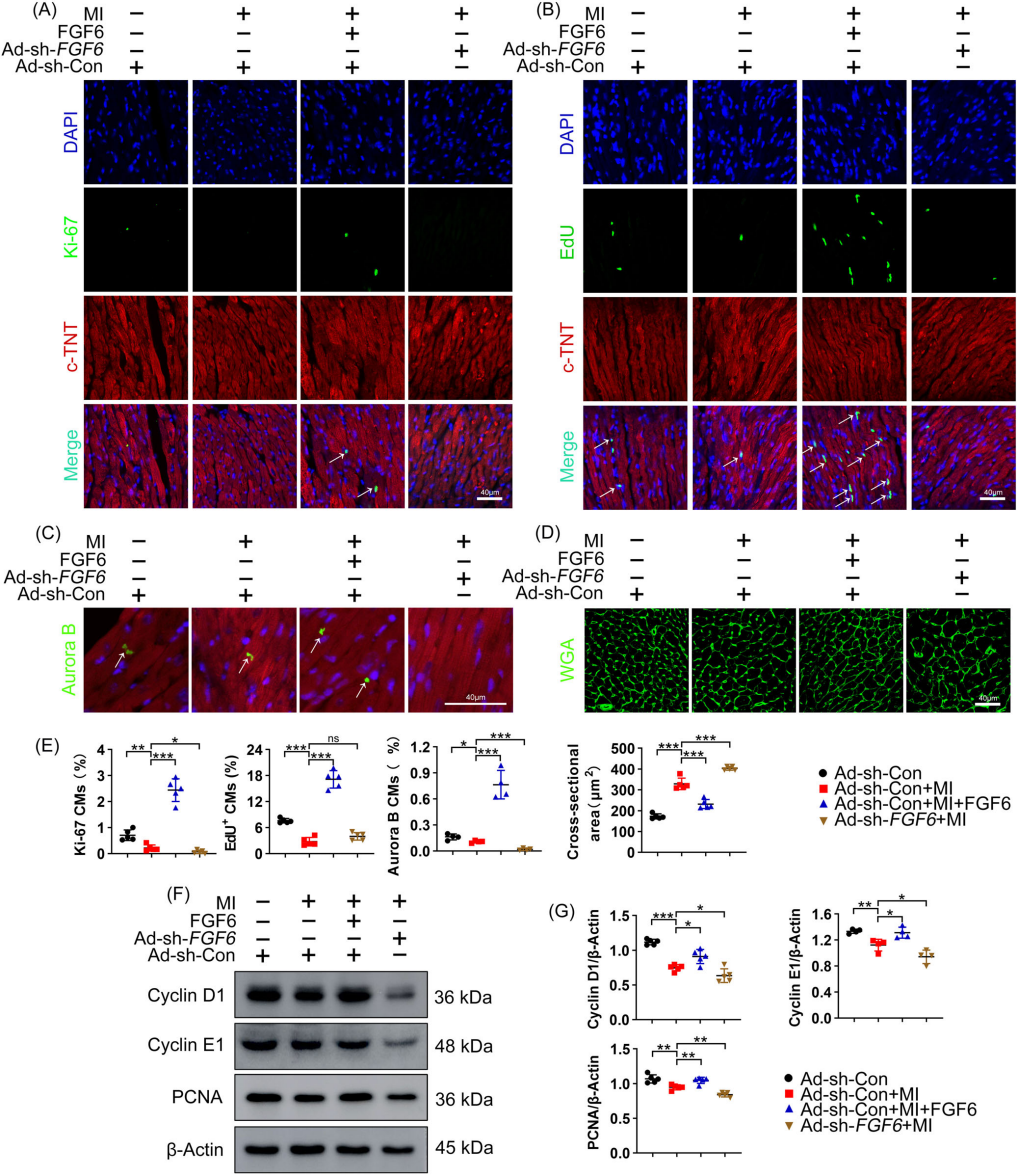
FGF6 promotes CMs cell cycle re‐entry in vivo. (A) Representative immunofluorescence (green for Ki‐67, red for cTNT, blue for DAPI) and quantitative analysis the number of Ki‐67 positive cardiomyocytes in different group mice heart sections, white arrow indicates positive cell. Scale bar =40 μm. *n* = 5 per group. (B) Representative immunofluorescence (green for EdU, red for cTNT, blue for DAPI) and quantitative analysis the number of EdU positive cardiomyocytes in different group mice heart sections, white arrow indicates positive cell. Scale bar =40 μm. *n* = 5 per group. (C) Representative immunofluorescence (green for Aurora B, red for cTNT, blue for DAPI) and quantitative analysis the number of Aurora B positive cardiomyocytes in different group mice heart sections, white arrow indicates positive cell. Scale bar = 40 μm. *n* = 4 per group. (D) WGA staining was performed to detect cardiomyocytes cross sectional areas in different group mice heart sections. Scale bar = 40 μm. *n* = 5 per group. (E) Quantitative analysis the number of Ki‐67 positive cardiomyocytes in (A), the number of EdU positive cardiomyocytes in (B), the number of Aurora B positive cardiomyocytes in (C) and the cardiomyocytes cross sectional areas in (D). (F) Western blot was performed to determine the protein levels of Cyclin D1, Cyclin E1 and PCNA in the heart of different group mice. *n* >4 per group. (G) Quantitative analysis the expressions of Cyclin D1, Cyclin E1 and PCNA in (E). Data represent means ± SEM. Two‐tailed Student's *t* test. *p <0.05, **p <0.01, ***p <0.001

To confirm the stimulatory effects of FGF6 on cardiac repair, 8‐week‐old mice were intraperitoneally injected with recombinant human FGF6 protein, and the cardiac reparative potential was examined 2 weeks after coronary artery ligation. FGF6 treatment greatly increased the numbers of Ki‐67‐, EdU‐ and Aurora B positive cells relative to MI mice (Figure 3A–C,E). Meanwhile, FGF6 treatment decreased the cross‐sectional area of CMs (Figure 3D,E) which reflects their proliferative potential. These results indicate that FGF6 treatment triggers the CMs cell cycle re‐entry and promotes cardiac repair in adult mice after MI. Furthermore, the levels of cell cycle‐related proteins, including Cyclin D1 and Cyclin E1, which promote progression from G1 to S phase, and of proliferating cell nuclear antigen (PCNA), a maker of proliferating cells, were decreased in hearts of MI mice, and these decreases were largely reversed by FGF6 treatment (Figure 3F). In addition, to evaluate the contribution of FGF6, the infarction area was infected with Ad‐sh‐*FGF6* in MI mice. Knockdown of FGF6 by Ad‐sh‐*FGF6* decreased the numbers of Ki‐67‐, EdU‐ and Aurora B positive cells in comparison with Ad‐sh‐Con infected MI mice (Figure 3A–E). These results suggest that injection of human recombinant FGF6 protein promotes cardiac repair after MI and contributes CMs cell cycle re‐entry. By contrast, silencing of FGF6 inhibits CMs cell cycle re‐entry and therefore obstructs cardiac repair in mice after MI.

**FIGURE 4:**
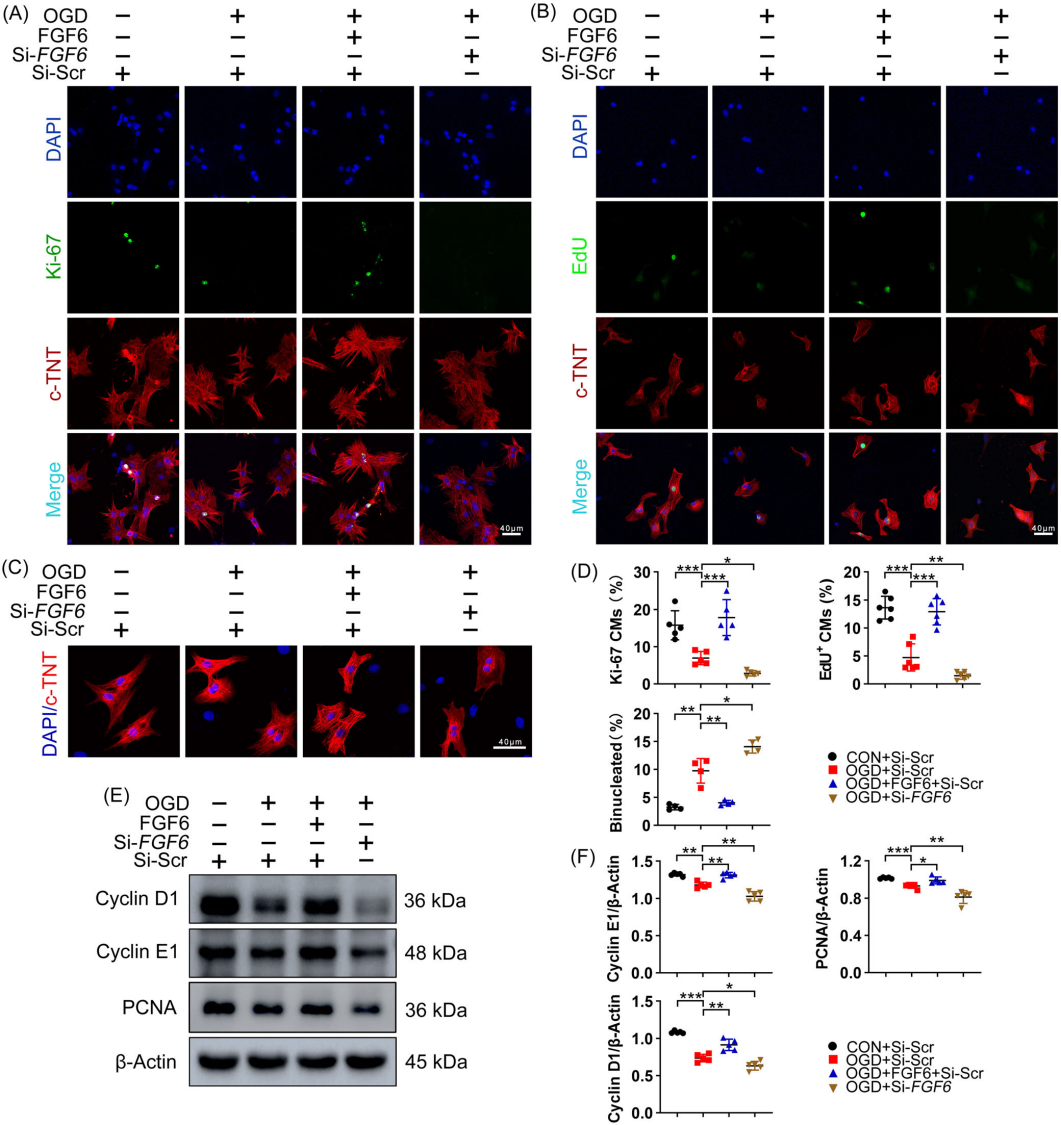
FGF6 promotes NRCMs cell cycle re‐entry in vitro. (A) Representative immunofluorescence (green for Ki‐67, red for cTNT, blue for DAPI) analysis the number of Ki‐67 positive NRCMs in different group. *n* = 5 per group. (B) Representative immunofluorescence (green for EdU, red for cTNT, blue for DAPI) analysis the number of EdU positive NRCMs in different group. Scale bar = 40 μm. *n* = 6 per group. (C) Representative immunofluorescence (red for cTNT, blue for DAPI) and quantitative analysis the number of binucleated NRCMs in different group. Scale bar = 40 μm. *n* = 4 per group. (D) Quantitative analysis the number of Ki‐67 positive NRCMs in (A), the number of EdU positive NRCMs in (B) and the number of binucleated NRCMs in (C). (E) Western blot was performed to determine the protein levels of Cyclin D1, Cyclin E1 and PCNA in NRCMs. *n* = 5 per group. (F) Quantitative analysis the expressions of Cyclin D1, Cyclin E1 and PCNA in (E). Data represent means ± SEM. Two‐tailed Student's *t* test. *p <0.05, **p <0.01, ***p <0.001

Furthermore, to obtain in vitro evidence for the beneficial role of FGF6 in cardiac repair, NRCMs were subjected to OGD in order to mimic ischemic injury of CMs in vitro. Immunofluorescence staining showed that after OGD for 6 h, proliferation potential was decreased, as reflected by decreased EdU and Ki‐67 staining (Figure 4A,B). Notably, immunofluorescence staining of c‐TNT showed that OGD obviously increased the number of binucleated cells (Figure 4C,D), which further confirmed their relatively low proliferative capacity. Immunoblot analysis demonstrated that OGD decreased the levels of Cyclin D1, Cyclin E1, and PCNA (Figure 4E,F). As expected, co‐treatment with FGF6 increased the numbers of Ki‐67‐ and EdU‐positive cells (Figure 4A,B), decreased the number of binucleated cells, and increased the protein levels of Cyclin D1, Cyclin E1, and PCNA (Figure 4C,E).

To further investigate the stimulatory effect of FGF6 on contribution to CMs cell cycle re‐entry, FGF6 was conditionally silenced in NRCMs by *FGF6*‐targeting siRNA (si‐*FGF6*) transfection. OGD had a greater detrimental effect on cell cycle re‐entry of these cells, as reflected by the more serious reduced numbers of Ki‐67‐ and EdU‐positive cells (Figure 4A,B), and by the more significant decreased levels of Cyclin D1, Cyclin E1, and PCNA (Figure 4E,F).

### 3.4 FGF6 contributes NRCMs cell cycle re‐entry by inhibiting the Hippo pathway

**FIGURE 5:**
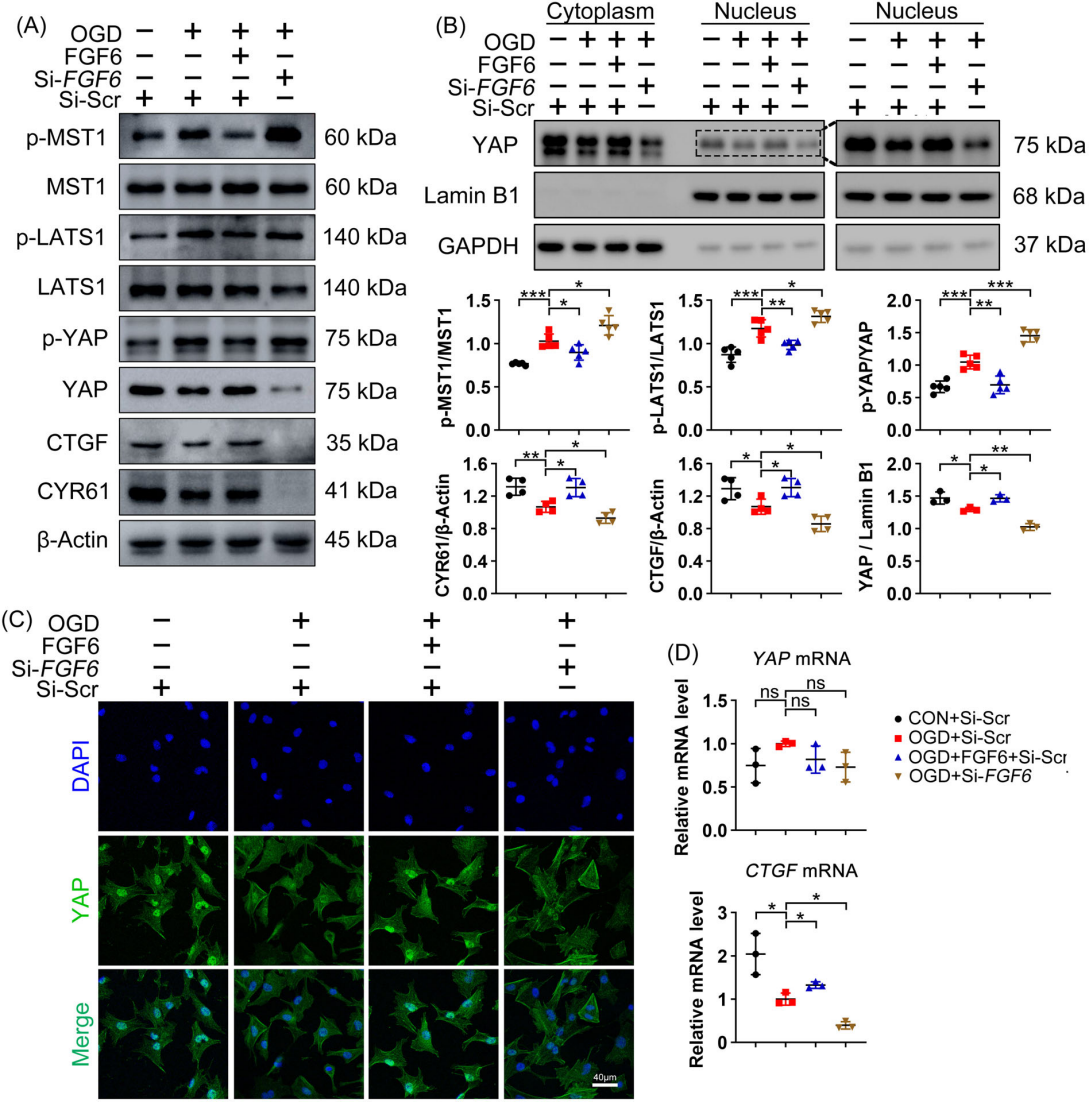
FGF6 inhibits activation of the Hippo pathway in NRCMs after OGD. (A) Western blot was performed to determine the protein levels of p‐MST1, MST1, p‐LATS1, LATS1, p‐YAP, YAP, CTGF and CYR61 in NRCMs in different group. *n* >4 per group. (B) Western blot analysis the subcellular localization of YAP in NRCMs in different group. Quantitative analysis of expressions of p‐MST1, MST1, p‐LATS1, LATS1, p‐YAP, YAP, CTGF and CYR61 in (A), quantitative analysis of expressions of YAP in nuclear extracts. (C) Representative immunofluorescence (green for YAP, blue for DAPI) analysis subcellular localization of YAP in NRCMs in different group. *n* = 5 per group. (D) RT‐PCR analysis of the mRNA levels of *YAP* and *CTGF* in NRCMs in different group. *n* = 3 per group. Data represent means ± SEM. Two‐tailed Student's *t* test. *p <0.05, **p <0.01, ***p <0.001

The Hippo/YAP pathway plays a critical role in cardiac repair. Thus, we investigated whether the cardioprotective effect of FGF6 is dependent on Hippo signalling. Immunoblotting showed that OGD decreased the protein level of YAP and increased phosphorylation of YAP (Figure 5A). Meanwhile, OGD also elevated the phosphorylation levels of MST1 and LATS1, other components of the Hippo pathway. By contrast, FGF6 treatment largely counteracted OGD induced activation of Hippo signalling, as reflected by decreased phosphorylation of YAP and its upstream kinases LATS1 and MST1. In addition, the elevated level of p‐YAP under OGD conditions was further increased by si‐*FGF6* treatment, and the total YAP protein level was the lowest between all the groups (Figure 5A). To our interesting, the mRNA level of *YAP* did not significantly differ between the groups (Figure 5D), indicating that FGF6 affects the level of YAP by blocking its degradation. Overall, these results indicate that FGF6 increases the level of YAP protein in CMs by inhibiting OGD induced activation of the Hippo pathway.

To investigate the role of YAP in ischemic heart repair, the level of YAP was measured in the nuclear fraction. The nuclear level of YAP was lower in the OGD treated group than in the control group and was further decreased by si‐*FGF6* treatment, while FGF6 treatment significantly counteracted the negative effect of OGD on nuclear accumulation of YAP (Figure 5B). Moreover, immunofluorescence staining showed that FGF6 treatment promoted nuclear translocation of YAP (Figure 5C). Next, we analysed the *CTGF*, a target gene of YAP, by RT‐PCR to confirm the transcriptional regulatory effect of YAP. As expected the mRNA level of *CTGF* correlated with nuclear accumulation of YAP (Figure 5D). In addition, western blotting provided evidence that FGF6 treatment promoted expression of CTGF and CYR61 via YAP (Figure 5A).

In summary, these results suggest that FGF6 inhibits the Hippo pathway and subsequently facilitates nuclear accumulation of YAP to activate downstream effector, and thereby promotes CMs cell cycle re‐entry under ischemic conditions.

### 3.5 FGF6 promotes cardiac repair by inhibiting the Hippo pathway in vivo

**FIGURE 6:**
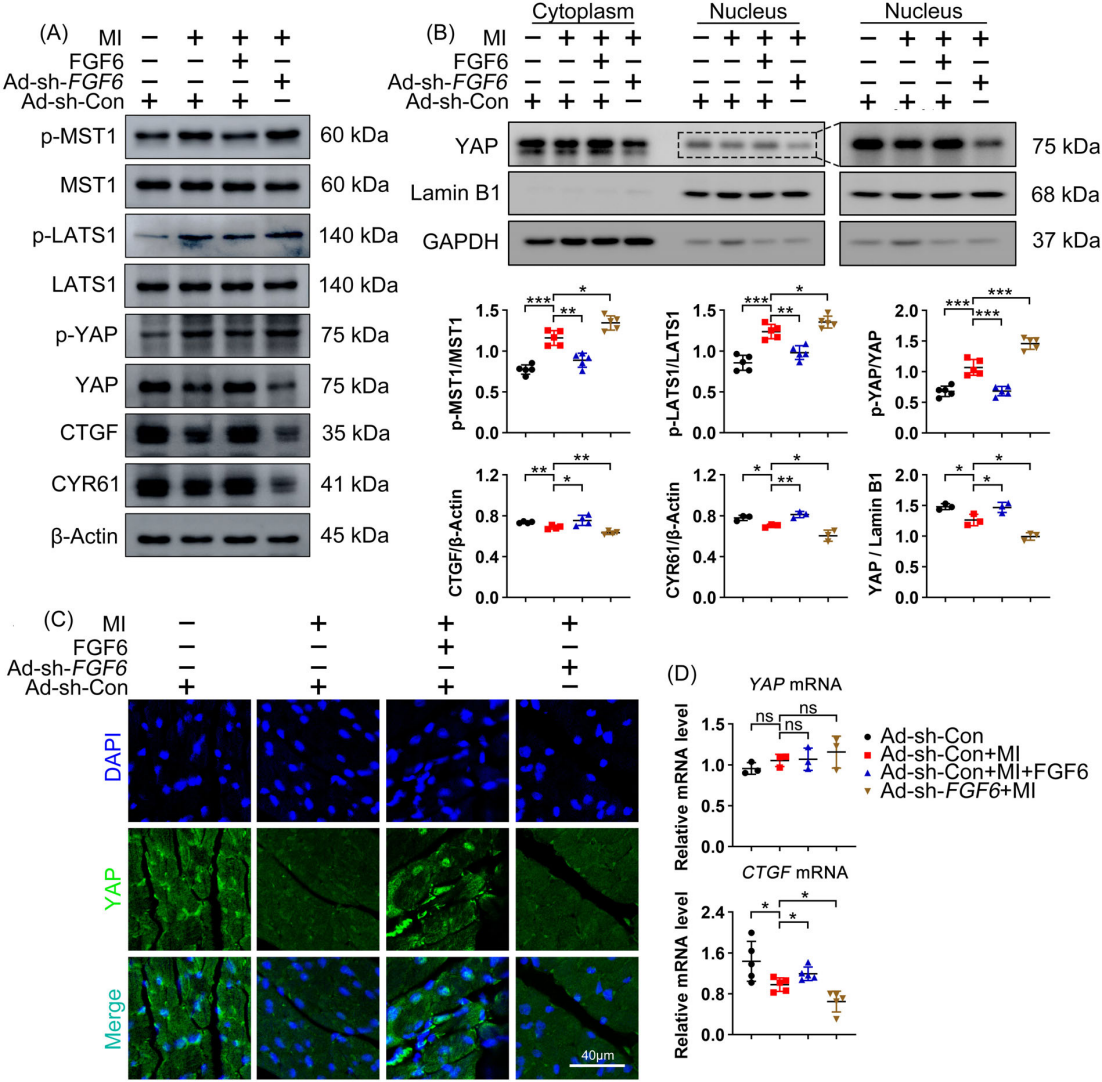
FGF6 inhibits activation of the Hippo pathway in CMs after MI. (A) Western blot was performed to determine the protein levels of p‐MST1, MST1, p‐LATS1, LATS1, p‐YAP, YAP, CTGF and CYR61 in the heart from different group mice. *n* >3 per group. (B) Western blot analysis the subcellular localization of YAP in the heart from different group mice. *n* = 3 per group. Quantitative analysis of expressions of p‐MST1, MST1, p‐LATS1, LATS1, p‐YAP, YAP, CTGF, and CYR61 in (A), quantitative analysis of expressions of YAP in nuclear extracts. (C) Representative immunofluorescence (green for YAP, blue for DAPI) analysis subcellular localization of YAP in the heart section from different group mice. (D) RT‐PCR analysis of the mRNA levels of *YAP* and *CTGF* in the heart from different group. *n* >3 per group; *n* = 5 per group. Data represent means ± SEM. Two‐tailed Student's *t* test. *p <0.05, **p <0.01, ***p <0.001

To confirm the influence of FGF6 on myocardial injury in vivo, wild‐type mice were subjected to MI by permanently ligating the left descending coronary artery. Whole ventricle analysis showed that phosphorylation of MST1, LATS1, and YAP were higher in MI than in sham‐operated mice, consistent with our in vitro finding that MST1 and LATS1 were activated after MI, along with elevated phosphorylation and subsequent nuclear exclusion of YAP and downregulation of CTGF and CYR61. Meanwhile, activation of the Hippo pathway in MI mice was aggravated by Ad‐sh‐*FGF6* mediated knockdown of FGF6, but was largely counteracted by administration of FGF6 (Figure 6A). Next, we sought to determine the subcellular distribution of YAP in response to different treatments after MI. Western blot analysis of fractionated CMs protein samples (Figure 6B). Immunofluorescence staining showed that nuclear YAP accumulation was decreased in MI mice and further reduced by knockdown of FGF6, but was remarkably elevated by FGF6 administration (Figure 6C). The mRNA level of *CTGF* showed a similar trend to the protein level of CTGF observed by western blotting, while the mRNA level of *YAP* did not differ between the groups (Figure 6D). Overall, these results imply that FGF6 blocks MI induced the activation of the Hippo pathway and subsequent nuclear exclusion of YAP.

### 3.6 FGF6 promotes cardiac repair through YAP

**FIGURE 7:**
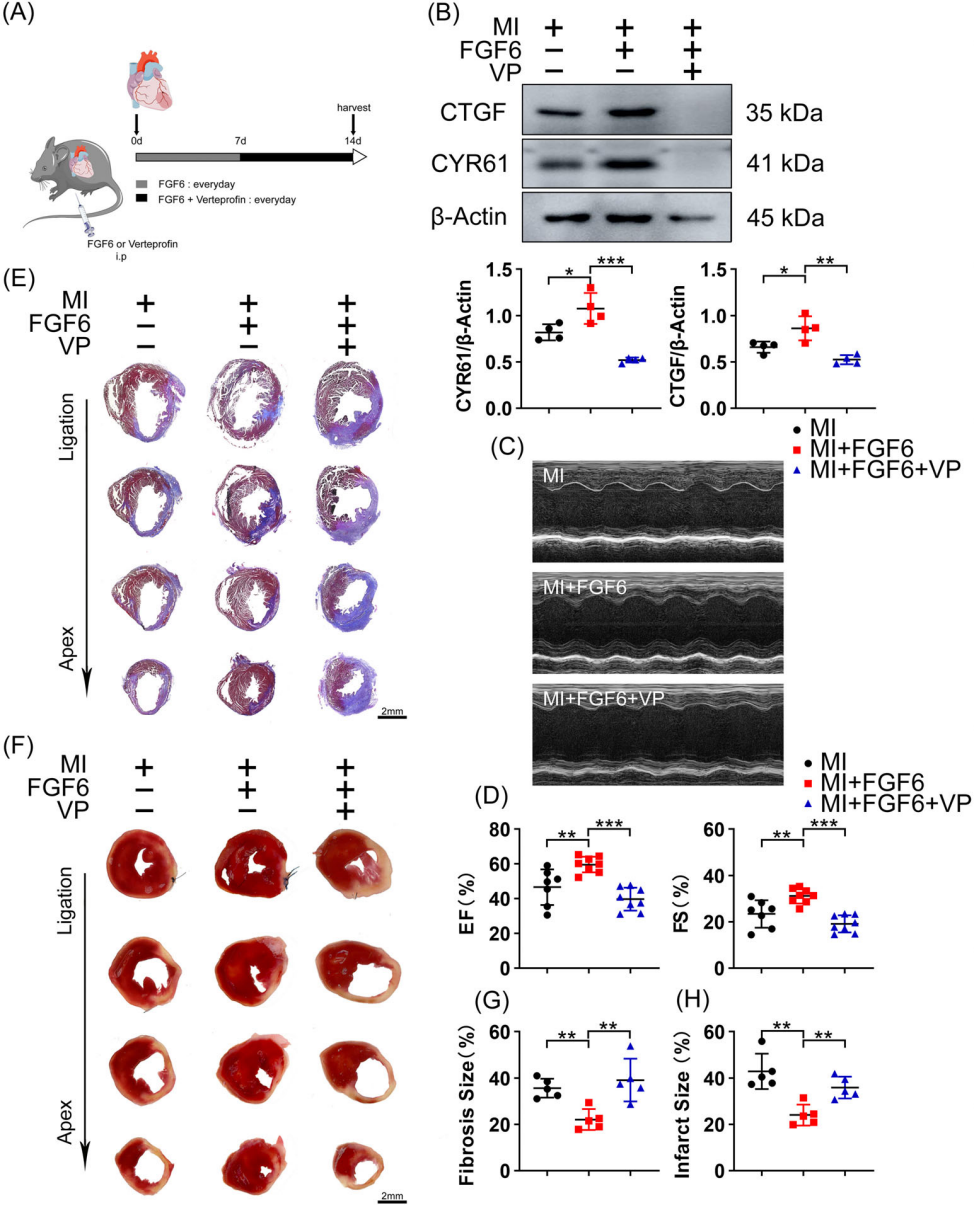
FGF6 protects cardiac function via YAP. (A) Schematic diagram demonstrates the animal experiment design. (B) Western blot was performed and quantitatively analysed to determine the protein levels of CTGF, CYR61 in the hearts of different group mice. *n* = 4 per group. (C) Representative M‐mode echocardiographic recording obtained from different group mice. *n* >7 per group. (D) Quantitative analysis of LV ejection fraction (EF), fractional shortening (FS). (E) Masson trichrome staining was performed to detect cardiac fibrosis area of different group mice. Scale bar = 2 mm. *n* = 5 per group. (F) TTC staining was performed to detect cardiac ischemic area of different group mice. Scale bar = 2 mm. *n* = 5 per group. (G) Quantitative analysis of cardiac fibrosis area in (E). (H) Quantitative analysis of cardiac ischemic area in (F). Data represent means ± SEM. Two‐tailed Student's *t* test. *p <0.05, **p <0.01, ***p <0.001

To clarify whether FGF6 promotes proliferation of CMs by inhibiting the Hippo pathway, YAP activity was blocked by treatment with VP which blocks binding of YAP to TEAD. Inhibition of YAP activity by VP largely counteracted the FGF6 induced elevation of CTGF and CYR61 expression (Figure 7A,B). Meanwhile, VP treatment blocked the cardioprotective effect of FGF6, as assessed by measuring EF and FS (Figure 7C,D). VP treatment largely abolished the FGF6 induced decreases in the area of cardiac fibrosis and infarct size measured by Masson's and TTC staining (Figure 7E–H). In addition, VP treatment abrogated the FGF6 induced restoration of the levels of Cyclin D1, Cyclin E1, and PCNA (Figures S2A and S3A) and the numbers of Ki‐67‐ and EdU‐positive CMs (Figures S2B–D and S3B–D) in vitro and in vivo. Together, these data suggest that the effects of FGF6 on CMs cell cycle re‐entry and cardiac repair under ischemic conditions are partially mediated by YAP.

### 3.7 FGF6 regulates the Hippo pathway via ERK1/2

**FIGURE 8:**
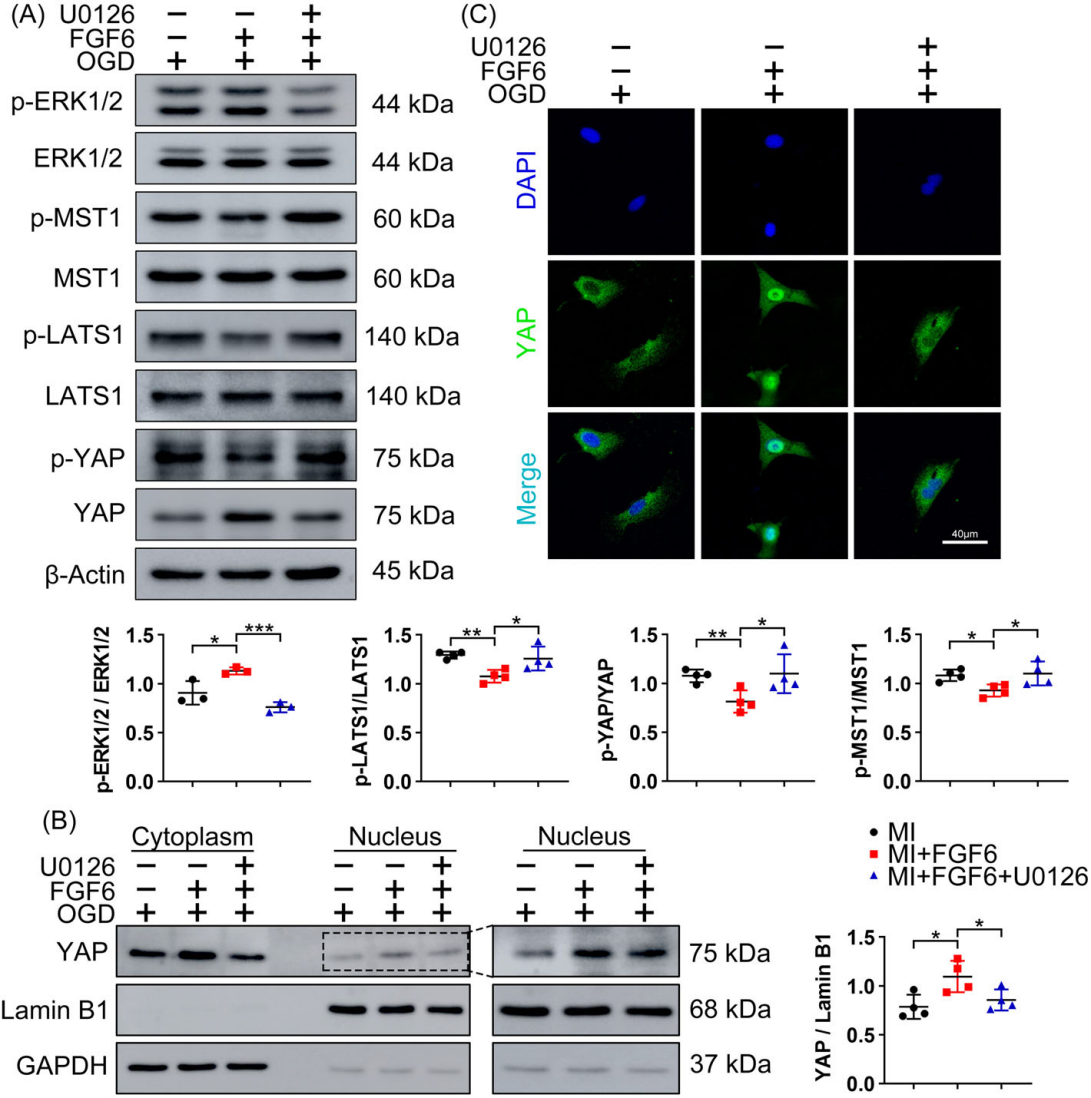
FGF6 inhibits the Hippo pathway via ERK1/2. (A) Western blot was performed to determine the protein levels of p‐ERK1/2, ERK1/2, p‐MST1, MST1, p‐LATS1, LATS1, p‐YAP and YAP in NRCMs in different group. Quantitative analysis of expressions of p‐MST1, MST1, p‐LATS1, LATS1, p‐YAP, YAP, CTGF and CYR61. *n* >3 per group. (B) Western blot analysis the subcellular localization of YAP in NRCMs in different group. *n* = 3 per group. Quantitative analysis of expressions of YAP in nuclear extracts. (C) Representative immunofluorescence (green for YAP, blue for DAPI) analysis subcellular localization of YAP in NRCMs in different group. *n* = 5 per group. Data represent means ± SEM. Two‐tailed Student's *t* test. *p <0.05, **p <0.01, ***p <0.001

Extracellular signal regulated kinase 1/2 (ERK1/2) is widely reported to regulate the Hippo signalling pathway. To clarify the role of ERK1/2 in FGF6‐mediated cardioprotection and its relationship with the Hippo pathway, we blocked activation of ERK1/2 using 30 μΜ U0126 (Figure 8A). The suppression effect of FGF6 on OGD induced activation of Hippo signalling, as reflected by decreased phosphorylation of MST1, LATS1, and YAP, and by subsequent nuclear accumulation of YAP, was largely abolished in the presence of U0126 (Figure 8A,B). Meanwhile, immunofluorescence staining further confirmed that inhibition of ERK1/2 decreased FGF6‐mediated nuclear localization of YAP (Figure 8C). Also, FGF6 facilitated elevation cell cycle protein Cyclin D1 Cyclin E1 and PCNA were reversed by U0126 treatment. (Figure S4A) Over all these results confirm that FGF6 elicits cardioprotective effects by reducing MI‐triggered activation of the Hippo pathway via activating ERK1/2.

## 4 DISCUSSION

**FIGURE 9:**
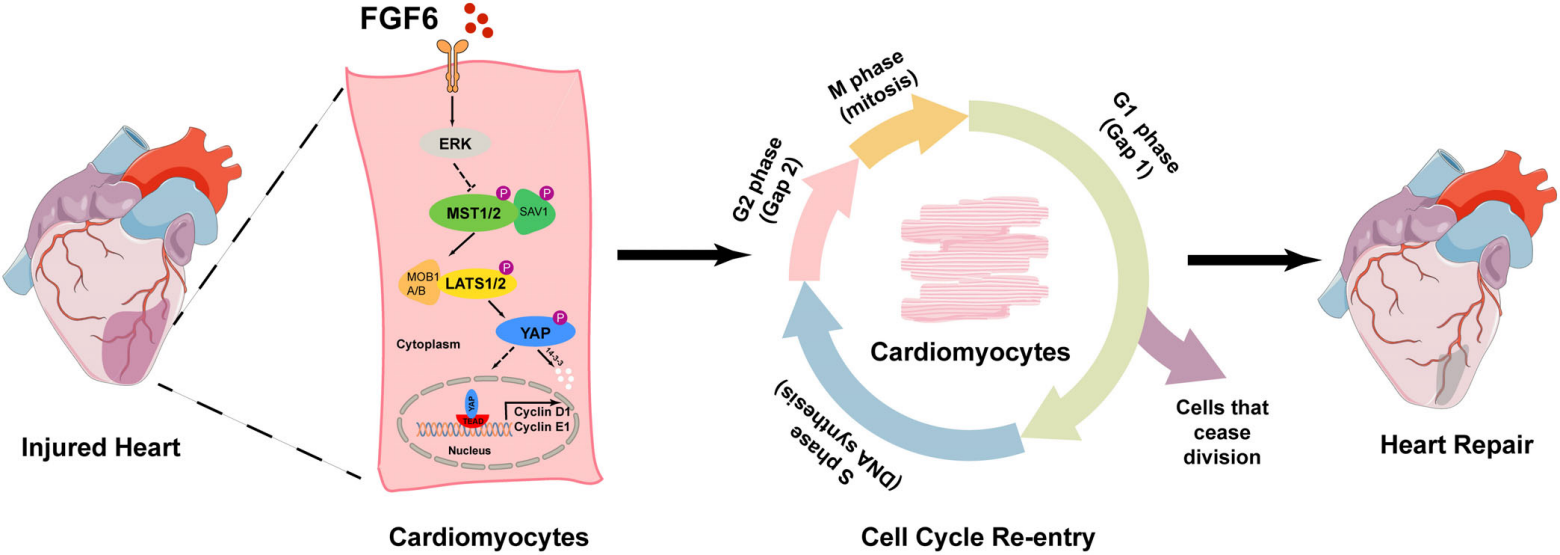
Schematic illustration of the protective effects of FGF6 on cardiomyocytes under ischemic conditions. Myocardial infarction would cause the loss of a huge number of cardiomyocytes. In this study, we revealed that FGF6 restrains the Hippo pathway via ERK, and promotes YAP nuclear translocation then increase cardiomyocytes cell cycle re‐entry and cardiac repair

In this study, we revealed that FGF6 protects against MI injury by inhibiting the Hippo pathway. FGF6 promotes nuclear accumulation of YAP and thereby, facilitates CMs cell cycle re‐entry and cardiac repair (Figure 9). These findings provide new clues and ideas for developing potential methods to treat MI and for promoting myocardial repair.

During MI, many CMs die after oxygen deprivation, resulting in progression of life‐threatening heart failure.^24^ Although CMs could proliferate to generate new CMs in the adult heart, their slow turnover rate is insufficient to compensate for the significant cell loss after MI.^25^, ^26^ Accordingly, we are exploring ways to improve the efficiency of CMs proliferation. Several lines of evidence indicate that CMs are not all equivalent and their proliferation is not randomly distributed. For instance, mononucleated CMs were reported to have a greater proliferative potential than multinucleated CMs, while pathological enlargement of cells reduces the likelihood of cell proliferation.^27^, ^28^ In this study, we found that FGF6 increased expression of Cyclin D1 and Cyclin E1, which are important to promote the transition from G1 to S phase in CMs.^29^, ^30^, ^31^

FGF6 expression is stimulated after skeletal muscle injury and can promote skeletal muscle regeneration, whereas interference of FGF6 can induce a severe regeneration defect with enhanced fibrosis and myotube degeneration.^32^, ^33^, ^34^ FGF6 plays a similar role in repair following myocardial injury. In this study, we revealed that FGF6 expression was elevated in ischemic CMs and cardiac tissues. FGF6 treatment significantly improved cardiac function by promoting cardiac repair in mice with cardiac ischemia. To the best of our knowledge, this is the first study to explore the molecular mechanisms by which FGF6 functions in myocardial repair.

There is emerging evidence that the Hippo pathway is a key player in a wide range of biological processes during growth and development of tissues and organs.^12^ Elevated expression of YAP, which is negatively regulated by the Hippo pathway, contributes to cardiac repair. Under normal conditions, YAP is phosphorylated and thus sequestered in the cytoplasm by 14‐3‐3 and inhibited. When the Hippo pathway is inactivated, YAP is dephosphorylated and translocates into the nucleus, where it binds to TEAD transcriptional co‐activators and thereby stimulates gene expression, which contributes to cell survival, migration, and proliferation.^21^, ^35^ In this study, we demonstrated that FGF6 treatment elevated expression and nuclear accumulation of YAP in ischemic CMs, without altering the *YAP* mRNA level, suggesting that degradation of YAP was restrained.

We noticed that FGF6 could activate the mitogen‐activated protein kinase signalling pathway and promote activation of ERK1/2. ERK1/2 is one of the mitogen‐activated protein kinases^27^, ^34^ the key components of the reperfusion injury salvage kinase pathway, and plays an important role in protecting the myocardium from lethal ischemia–reperfusion injury. Constitutive activation of mitogen‐activated protein kinase 1 (CaMEK) promotes ERK1/2 expression, which is expected to protect the heart against ischemia–reperfusion injury.^25^, ^27^ The relationship between ERK1/2 and the Hippo pathway has been reported in tumour diseases.^36^ By using U0126, a specific ERK1/2 inhibitor, we showed that the cardioprotective effect of FGF6 via blockade of the Hippo pathway was primarily mediated by ERK1/2. Considering that treatment with VP, a specific YAP inhibitor, did not significantly change ERK1/2 activity in vitro or in vivo, we speculated that ERK1/2 functions upstream of the Hippo pathway. However, further exploration are still needed.

MI causes loss of a huge number of CMs. It is clinically treated mainly using interventional therapy, which cannot prevent cell death. Exploration of alternative drugs or strategies would be extremely valuable for management of MI patients. Meanwhile a growing number of results suggest that a strategy to promote CMs proliferation is most favourable to protect the heart during the process of regeneration.^37^, ^38^, ^39^, ^40^ Our study suggests that FGF6 is a powerful regulator to promote CMs cell cycle re‐entry and improve cardiac function after MI. And this study also demonstrates that FGF6 attenuates activation of the Hippo pathway via ERK1/2 and progression of cardiac ischemic injury. Although the effect of FGF6 on MI injury requires further investigation and subsequent clinical translation our study presents novel evidence that FGF6 is an effective candidate drug to treat MI.

## CONFLICT OF INTEREST

The authors declare no competing interests.

## AUTHOR CONTRIBUTION

Zhicheng Hu, Linlin Wang, Gen Chen, Yang Wang and Xueqiang Guan conceived, designed and supervised the study. Peng Chen, Yu Zhu, Zhicheng Hu, Zhenyu Hu researched the data. Yunjie Chen, Weijing You, Gen Chen, Lin Mei and Zhicheng Hu contributed to the discussion and design of the project. Zhicheng Hu, Yu Zhu, Xu Wang, Litai Jin, and Weitao Cong wrote the paper. Zhicheng Hu and Peng Chen are the guarantor of this work and, as such, had full access to all the data in the study and takes responsibility for the integrity of the data and the accuracy of the data analysis. All authors read and approved the final manuscript.

## Supporting information


**Data S1**: Supporting information

## Data Availability

Data supporting the findings of this study are available from the corresponding author upon reasonable request
