# Supplementary material for: FGF6 promotes cardiac repair after myocardial infarction by inhibiting the Hippo pathway
Source: Cell Prolif. 2022 Mar 30;55(5):e13221. doi: 10.1111/cpr.13221 (PMC9136516; doi:10.1111/cpr.13221)
Supplement: Supplementary file 1 — Data S1: Supporting information [file CPR-55-e13221-s001.doc]

**Supplementary information**

**FGF6 promotes cardiac repair after myocardial infarction by inhibiting the Hippo pathway**

Zhicheng Hu^1, 2†^, Peng Chen^1†^, Linlin Wang^3†^, Yu Zhu^4^, Gen Chen^2, 5^, Yunjie Chen^6^, Zhenyu Hu^2^, Lin Mei^2^, You Weijing^7^ Weitao Cong^2^, Litai Jin^2^, Xu Wang^2*^, Yang Wang^4*^ and Xueqiang Guan^1*^

**Author names and institutional affiliations**

^1^Department of Cardiology, The Second Affiliated Hospital and Yuying Children’s Hospital of Wenzhou Medical University, Wenzhou, 325027, P.R. China.

^2^School of Pharmaceutical Science, Wenzhou Medical University, Wenzhou, 325000, P.R. China.

^3^Children's Heart Center, Institute of Cardiovascular Development and Translational Medicine, The Second Affiliated Hospital and Yuying Children’s Hospital of Wenzhou Medical University, Wenzhou, 325027, P.R. China.

^4^Department of Histology and Embryology, Institute of Neuroscience, Wenzhou Medical University, Wenzhou, 325000, P.R. China.

^5^College of Pharmacy, Chonnam National University, Gwangju 500-757, Korea.

^6^Department of Pharmacy, Ningbo first Hospital, Ningbo, 315010, PR China.

^7^Ningbo College of Health Sciences, Ningbo 315211, PR China.

^†^ These authors contributed equally.

**
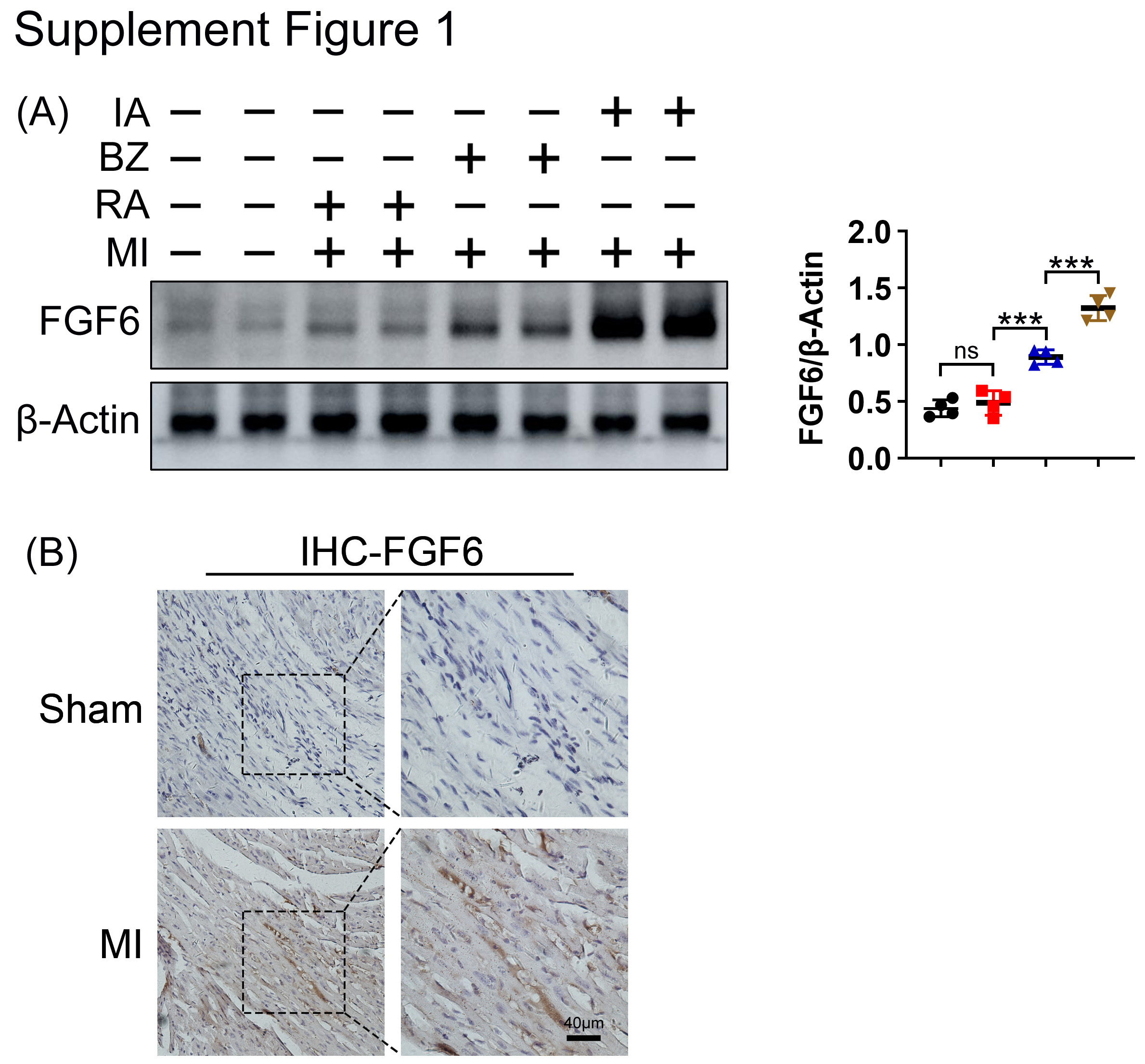
**

**Supplement Figure 1. FGF6 expression is increased in infarct area after MI.**

(A) Western blot was performed and quantitatively analyzed to determine the protein levels of FGF6 in the infarct area, border zone, remote area or sham-operated controls. n =4 per group. (B) Representative immumohistochemical staining analysis of FGF6 proteins in the heart tissues from MI (2 weeks) or sham. Scale bar =40 μm. Data represent means ±SEM. Two-tailed student’s t test. *** *P*<0.001.

**
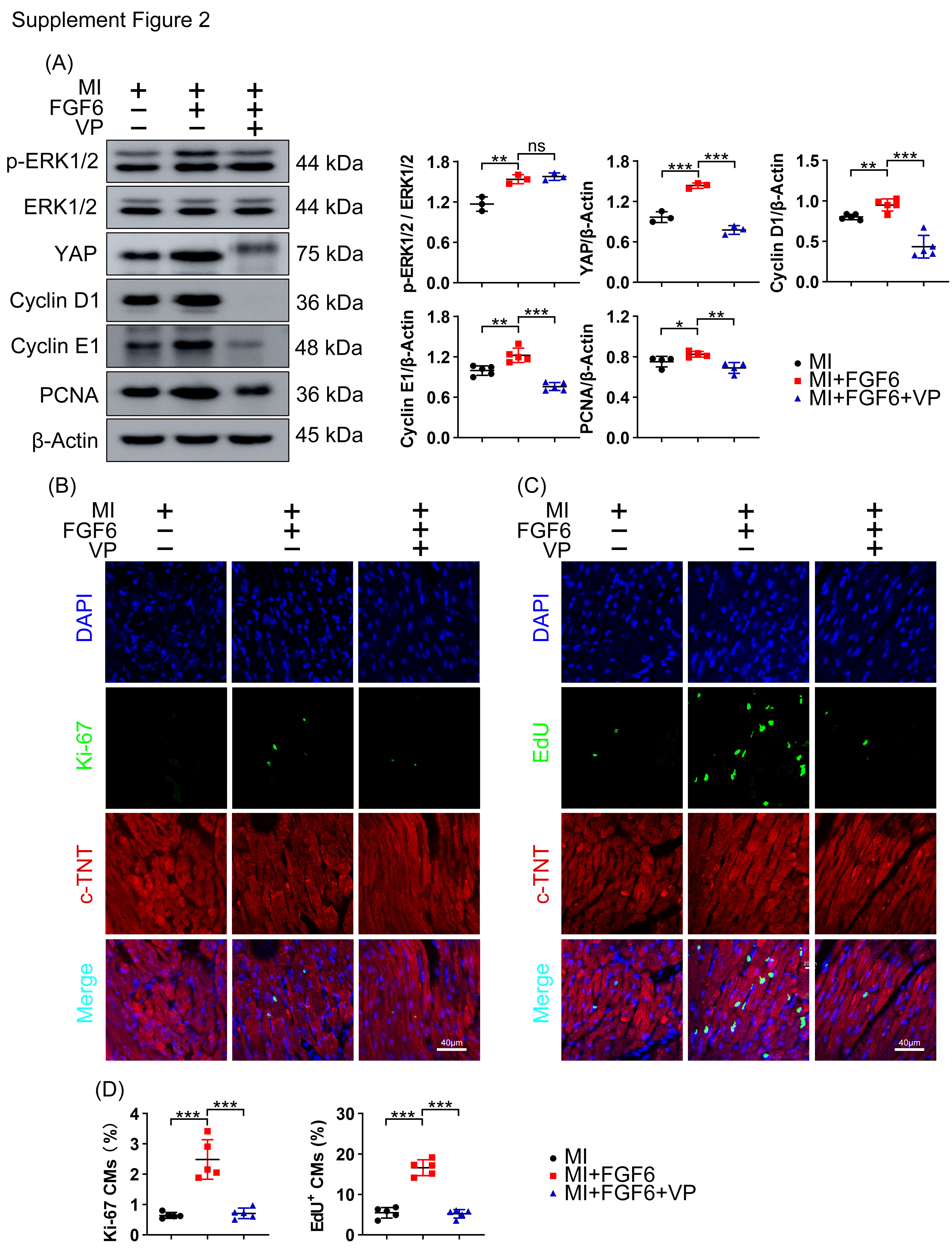
**

**Supplement Figure 2. FGF6 promotes cardiomyocytes cell cycle re-entry via YAP *in vivo*.**

(A) Western blot was performed and quantitative analysis to determine the protein levels of p-ERK1/2, ERK1/2, YAP, Cyclin D1, Cyclin E1 and PCNA in the heart of different group mice. n >3 per group. (B) Representative immunofluorescence (green for Ki-67, red for cTNT, blue for DAPI) and quantitative analysis the number of Ki-67 positive cardiomyocytes in different group mice heart sections. Scale bar =40 μm. n =5 per group. (C) Representative immunofluorescence (green for EdU, red for cTNT, blue for DAPI) and quantitative analysis the number of EdU positive cardiomyocytes in different group mice heart sections. Scale bar =40 μm. n =5 per group. (D) Quantitative analysis the number of Ki-67 positive cardiomyocytes in (B), the number of EdU positive cardiomyocytes in (C). Data represent means ±SEM, Two-tailed student’s t test. * *P*<0.05, ** *P*<0.01, ****P*<0.001.**
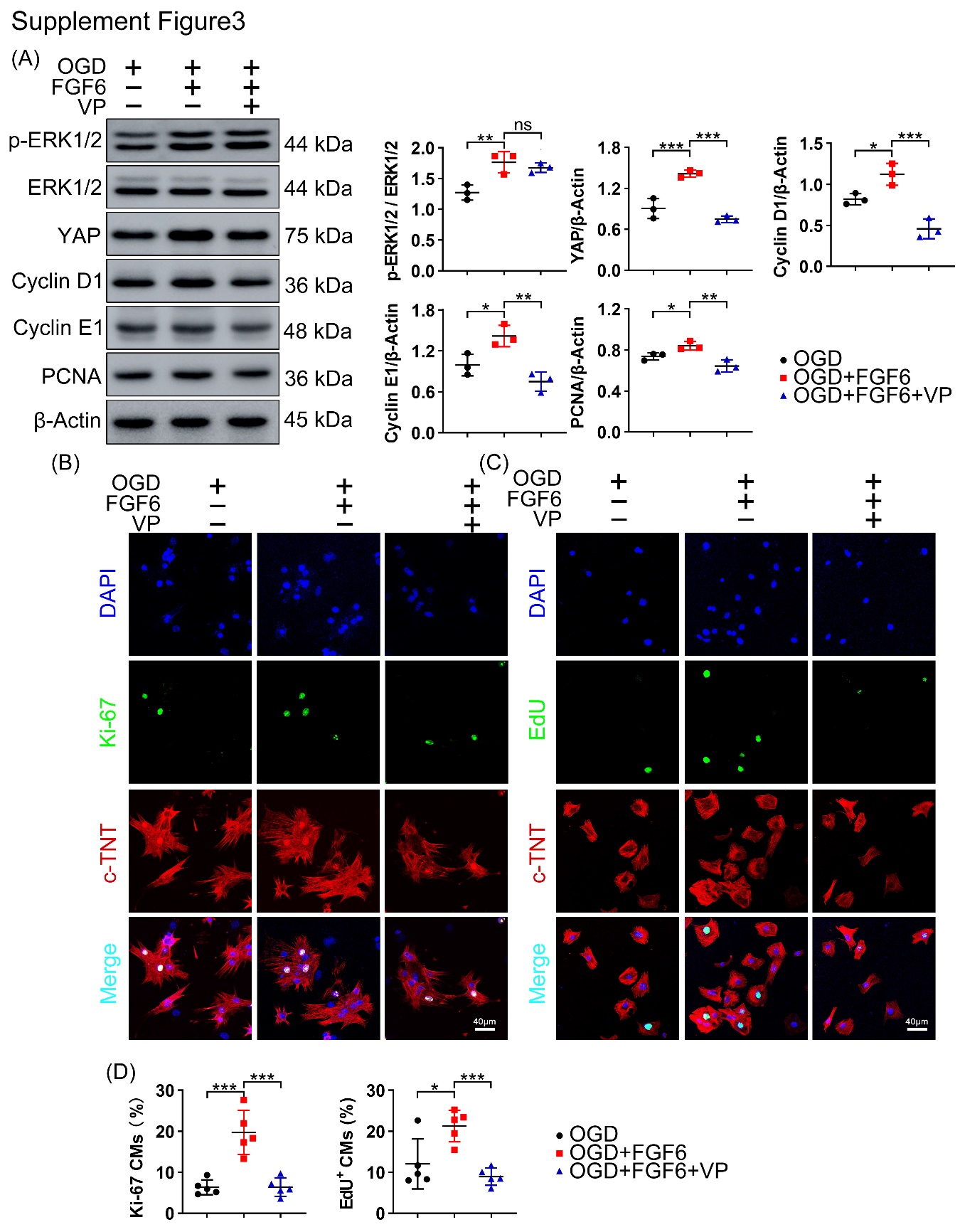
Supplement Figure 3. FGF6 promotes cardiomyocytes cell cycle re-entry via YAP *in vitro*.**

(A) Western blot was performed and quantitative analysis to determine the protein levels of p-ERK1/2, ERK1/2, YAP, Cyclin D1, Cyclin E1 and PCNA in NRCMs. n = 3 per group. (B) Representative immunofluorescence (green for Ki-67, red for cTNT, blue for DAPI) and quantitative analysis the number of Ki-67 positive NRCMs in different group. Scale bar =40 μm. n =5 per group. (C) Representative immunofluorescence (green for EdU, red for cTNT, blue for DAPI) and quantitative analysis the number of EdU positive NRCMs in different group. Scale bar =40 μm. n =5 per group. (D) Quantitative analysis the number of Ki-67 positive NRCMs in (B), the number of EdU positive NRCMs in (C). Data represent means ±SEM, Two-tailed student’s t test. * *P*<0.05, ** *P*<0.01, *** *P*<0.001.

**
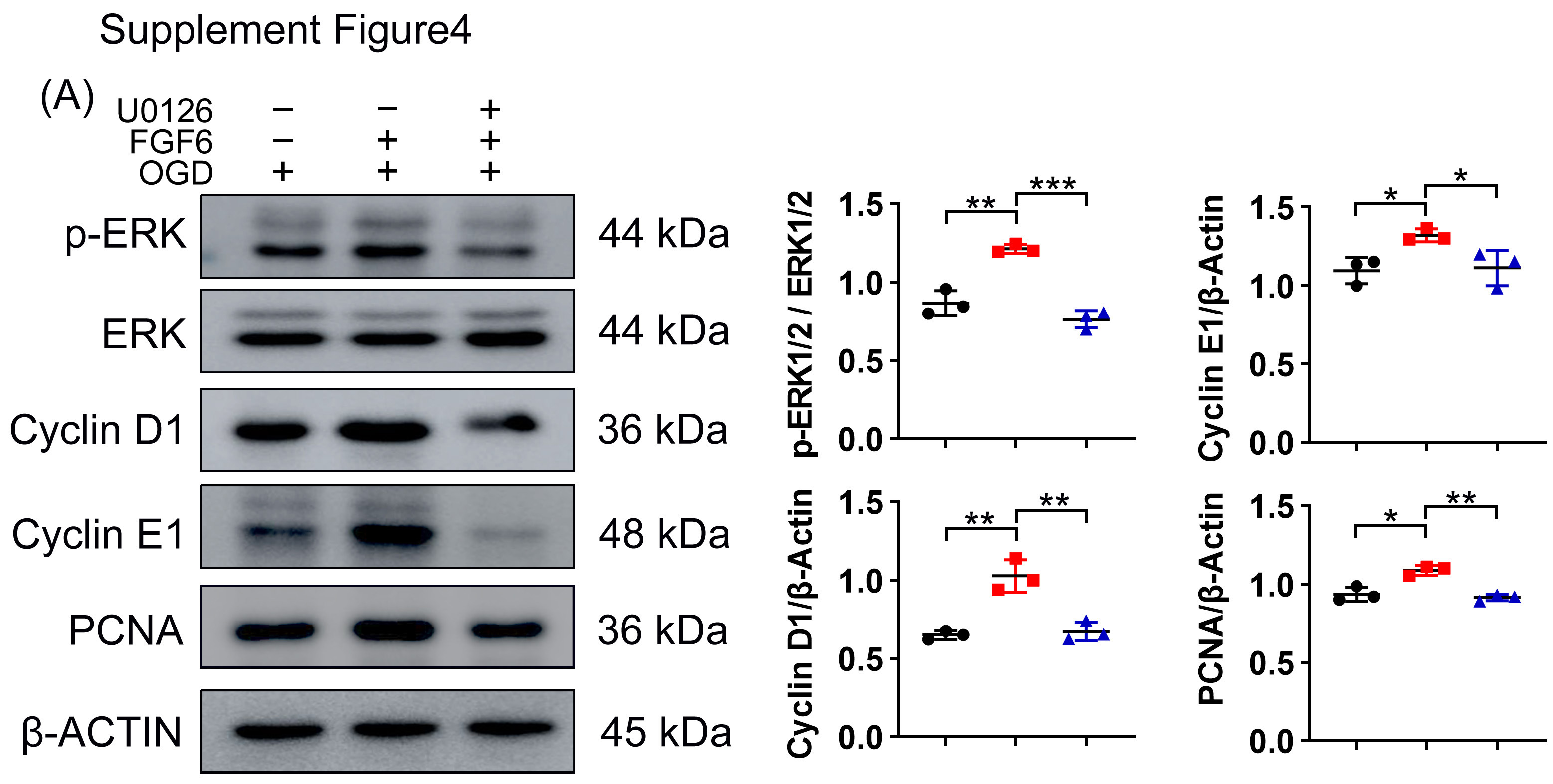
**

**Supplement Figure 4. FGF6 promotes cardiomyocytes cell cycle re-entry via ERK.**

(A) Western blot was performed and quantitative analysis to determine the protein levels of p-ERK1/2, ERK1/2, Cyclin D1, Cyclin E1 and PCNA in the heart of different group mice. n =3 per group. Data represent means ±SEM, Two-tailed student’s t test. * *P*<0.05, ** *P*<0.01, *** *P*<0.001.

**Supplementary Table 1. Detailed primers used in the RT-PCR.**

| Primer | Forward | Reverse |
| --- | --- | --- |
| *YAP (Rat)* | 5'- TTTGCCATGAACCAGAGGAT -3' | 5'- TATCTGCTGCTGCTGGTTTG -3' |
| *FGF6 (Rat)* | 5'- GGACGCACGAGGAGAACCCCT -3' | 5'- CTTGCATTCGTCCTGGAAGCTG -3' |
| *CTGF(Rat)* | 5'- GAGTCGTCTCTGCATGGTCA -3' | 5'- GCAGCCAGAAAGCTCAAACT- 3' |
| *GAPDH (Rat)* | 5'- ATCAAGAAGGTGGTGAAGCA -3' | 5'- AAGGTGGAAGAATGGGAGTTG -3' |
| *YAP (*Mouse*)* | 5'- ACCCTCGTTTTGCCATGAAC -3' | 5'- TGTGCTGGGATTGATATTCCGTA -3' |
| *FGF6 (Mouse)*  *CTGF(*Mouse*)* | 5'- CAGGCTCTCGTCTTCTTAGGC -3'  5'- CACCTAAAATCGCCAAGCCTG -3' | 5'- TTCACACCCGAAATCTCTCCA -3'  5'- AGTTCGTGTCCCTTACTTCCTG -3' |
| *GAPDH (Mouse)* | 5'- AGGTCGGTGTGAACGGATTTG -3' | 5'- TGTAGACCATGTAGTTGAGGTCA -3' |
